# Characterization of cellular transcriptomic signatures induced by different respiratory viruses in human reconstituted airway epithelia

**DOI:** 10.1038/s41598-019-48013-7

**Published:** 2019-08-07

**Authors:** Claire Nicolas de Lamballerie, Andrés Pizzorno, Julia Dubois, Thomas Julien, Blandine Padey, Mendy Bouveret, Aurélien Traversier, Catherine Legras-Lachuer, Bruno Lina, Guy Boivin, Olivier Terrier, Manuel Rosa-Calatrava

**Affiliations:** 1 Virologie et Pathologie Humaine - VirPath team, Centre International de Recherche en Infectiologie (CIRI), INSERM U1111, CNRS UMR5308, ENS Lyon, Université Claude Bernard Lyon 1, Université de Lyon, Lyon, 69008 France; 2 Viroscan3D SAS, Lyon, 69008 France; 3 VirNext, Faculté de Médecine RTH Laennec, Université Claude Bernard Lyon 1, Université de Lyon, Lyon, 69008 France; 4 Research Center in Infectious Diseases of the CHU de Quebec and Laval University, Quebec City, QC G1V 4G2 Canada

**Keywords:** Virus-host interactions, Influenza virus

## Abstract

Acute respiratory infections, a large part being of viral origin, constitute a major public health issue. To propose alternative and/or new therapeutic approaches, it is necessary to increase our knowledge about the interactions between respiratory viruses and their primary cellular targets using the most biologically relevant experimental models. In this study, we used RNAseq to characterize and compare the transcriptomic signature of infection induced by different major respiratory viruses (Influenza viruses, hRSV and hMPV) in a model of reconstituted human airway epithelia. Our results confirm the importance of several cellular pathways commonly or specifically induced by these respiratory viruses, such as the innate immune response or antiviral defense. A very interesting common feature revealed by the global virogenomic signature shared between hRSV, hMPV and influenza viruses is the global downregulation of cilium-related gene expression, in good agreement with experimental evaluation of mucociliary clearance. Beyond providing new information about respiratory virus/host interactions, our study also underlines the interest of using biologically relevant experimental models to study human respiratory viruses.

## Introduction

Acute respiratory infections (ARI) constitute a leading cause of acute illness worldwide and a major cause of death among young children, with nearly 2 million deaths per year^1–3^. Among a panoply of different viral and bacterial pathogens, respiratory viruses, such as influenza A and B viruses (IAV and IBV), respiratory syncytial virus (hRSV-A and hRSV-B), human metapneumovirus (hMPV-A and hMPV-B) or rhinoviruses (RV), represent the main etiologic agents of these infections^2,4,5^. In that regard, the limited or non-existent prophylactic and therapeutic arsenal available, coupled with the emergence of antiviral resistance, highlight the public health burden imposed by these respiratory pathogens. It appears therefore urgent to develop alternative and/or new therapeutic approaches, for which it is a necessary condition to increase our knowledge of the interactions between respiratory viruses and their primary cellular targets, namely respiratory epithelial cells.

The last decade has witnessed the development of high-throughput “omics” approaches that have contributed to deepen our understanding of the multiple levels of interplay between respiratory viruses and the host cell. Numerous studies have described the impact of infection on host gene expression *in vitro* or *in vivo*, mainly in the context of influenza viruses or hRSV and to a lesser extent for other respiratory viruses such hMPV^6–10^. For example, several mRNA profiling studies, including ours, have highlighted the role of NF-kB, p53, or MAPK cellular pathways in the context of influenza virus infection^11–13^. Nevertheless, only few comparative studies between different respiratory viruses have been so far described in the literature, although such approaches would be very informative to determine common and/or specific viral “fingerprints” that could provide valuable insight on potential antiviral immune response countermeasures and other pathogenesis mechanisms. Moreover, we still face the challenge of achieving increased physiologic relevance by coupling transcriptomic analyses with measurable physiological, immunological and/or virological data.

In a context of limited access to large panels of high-quality clinical samples, human reconstituted airway epithelial (HAE) models appear as biologically relevant and useful tools for studying infection at the level of primary target cells for respiratory viruses. For example, both in-house and commercially available (i.e. Mucilair™) HAE models, composed of human primary ciliated columnar cells, mucus-secreting goblet and basal cells cultivated at the air-liquid interface, have been successfully used to study viral infections and evaluate antivirals^14–17^. In that regard, the aim of the present study was to perform a comparative analysis of transcriptomic signatures of infection among different major respiratory viruses (Influenza, hRSV and hMPV) in HAE, in order to identify and experimentally validate both common and specific key cellular pathways. Altogether, our results confirmed the importance of several cellular pathways, such as the interferon signaling and cilium-related pathways, specifically or commonly deregulated upon infection by different respiratory viruses, which were further validated through transversal analyses such as quantification of secreted cytokines/chemokines. Beyond providing new data highlighting the interplay between respiratory viruses and the host cells, this study also underlines the interest of using biologically relevant models such as reconstituted HAE as a good compromise between *in vitro* immortalized cell lines and *ex-vivo*/*in-vivo* experimental models for the functional study of respiratory pathogens.

## Results

### Experimental setup and monitoring of viral infection in HAE

**Figure 1:**
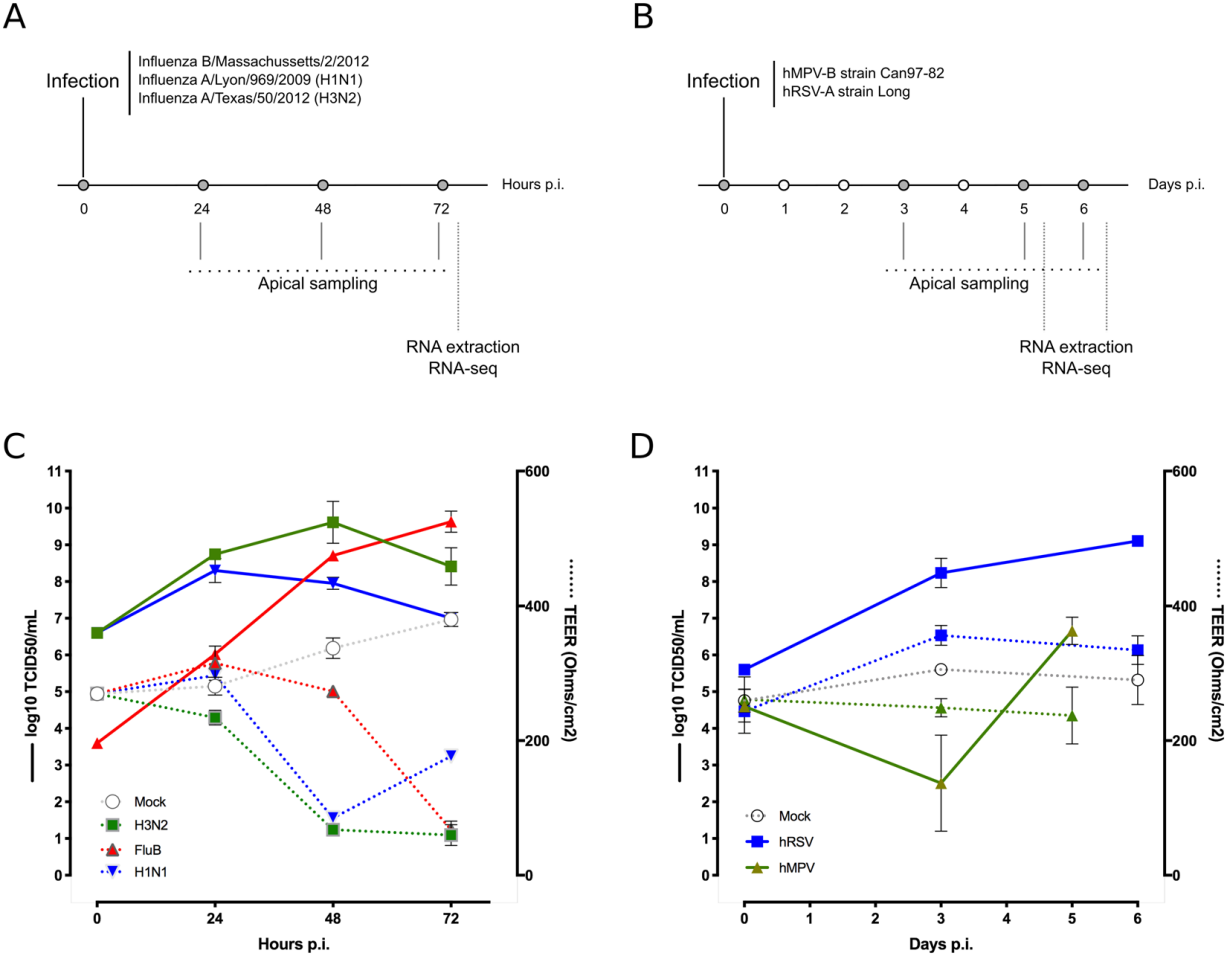
Viral infection in HAE: experimental protocol and biological monitoring results. (**A**,**B**) Apical poles were gently washed with 200 µL of OptiMEM medium. 150 µL of diluted viruses (Influenza B/Massachussets/2/2012, Influenza A/Lyon/969/2009, Influenza A/Texas/50/2012 in panel A and hMPV-B strain CAN97-82 or hRSV-A strain Long in panel (B) were added to the apical pole and incubated for 1 h at 37 °C. The infection is mimicked in the mock condition by adding virus-free OptiMEM medium. The viral suspension was then removed and apical poles were washed with OptiMEM. The OptiMEM medium was removed and air-liquid interface was restored. Kinetic samplings and measures were performed at the specified timing and after the last sampling, cells were lysed in RLT buffer and stocked at −80 °C before RNA extraction. (**C**,**D**) Membrane resistance between the apical and basal pole was measured at each time-point. The resulting trans-epithelial resistance (TEER in Ohms/cm^2^) is represented in dotted line and color-coded in accordance with infectious condition (mock in white, H3N2 in green, influenza B in red and H1N1 in blue in panel C and blue for hRSV and green for hMPV-B in panel D). Viral production at the apical pole was assessed after apical medium collection at the indicated time-points: 24-48-72hpi for influenza viruses and 3dpi and 5 or 6dpi for hRSV and hMPV. Viral titers indicated at T = 0 in correspond to the input viral load as validated by back-titration. For each time-point, MDCK (influenza virus) or LLC-MK2 cells (hRSV/hMPV) were incubated with serial dilutions from collected sample for the determination of viral titers. The specific parameters (infection protocol, MOI, time-points) previously optimized allow a precious and precise infection monitoring with appropriate viral replication (increasing titers) and a dropping mobility capacity of cilium.

**Figure 2:**
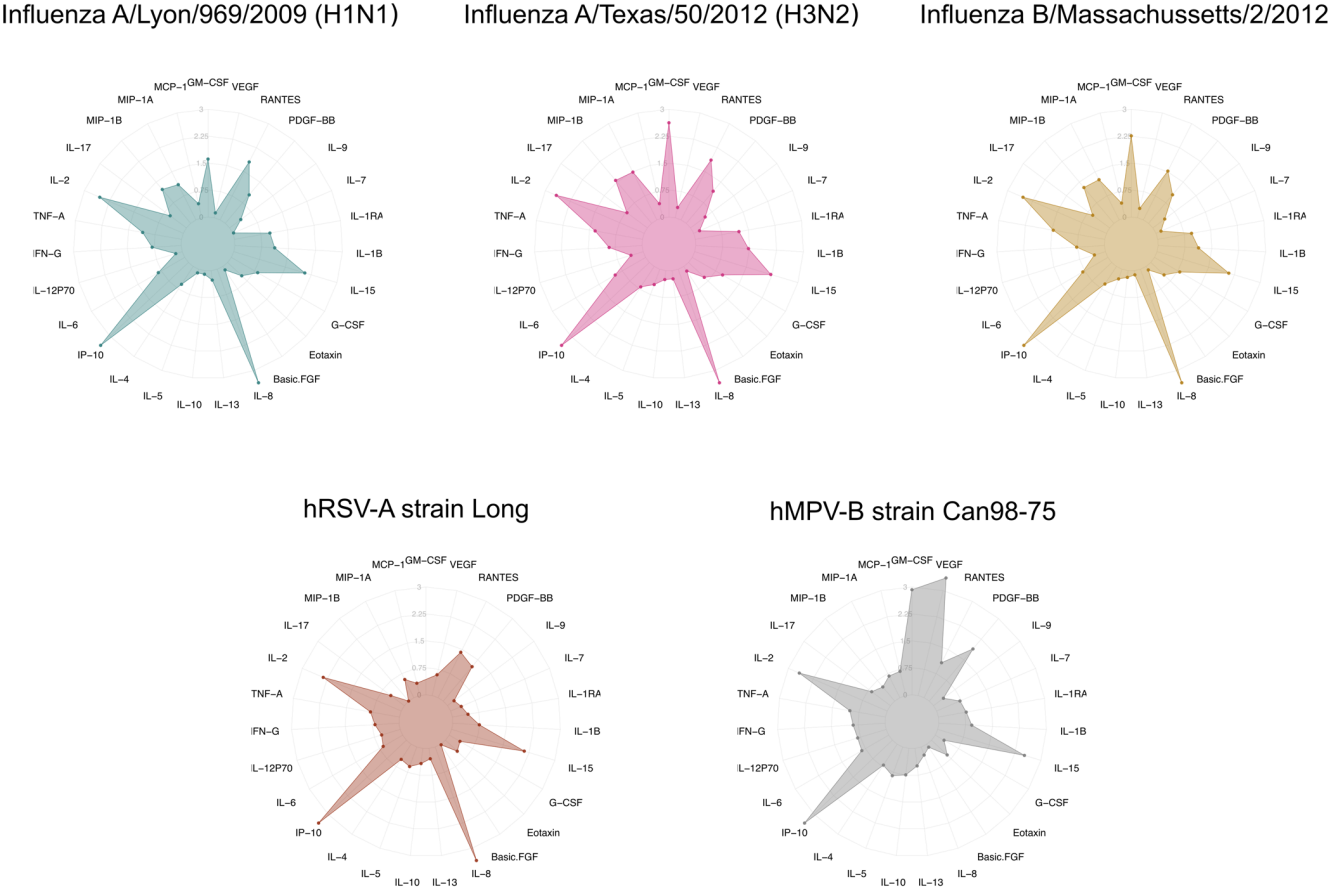
Comparison of immunity-related protein secretion profiles of infected airway epithelia. Chemokines and cytokines (FGF basic, Eotaxin, G-CSF, GM-CSF, IFN-γ, IL-1β, IL-8, IL-9, IL-10, IL-12, IL-13, IL-15, IL-17A, IP-10, MCP-1, MIP-1α, MIP-1β, PDGF-BB, RANTES, TNF-α and VEGF) harvested at the apical pole were quantified on a FLEXMAP 3D analyzer (Luminex, Austin, Texas, USA). Cytokine concentration values are represented in log10-transformation of the ratio (viral cytokine concentration) / (mock cytokine concentration). Because no signal could be assessed for GM-CSF, in the case for hRSV, this specific point was set at 0. Each radar chart accounts for the 27 cytokine/chemokine levels in each viral infection.

The initial step of our study was to produce high quality, reproducible, and physiologically relevant material in order to transversely analyze the common and distinct features of infection with major respiratory viruses. To that end, we performed a series of experimental infections in HAE using specific parameters (infection protocol, MOI, time-points) previously optimized according to both the literature and in-house pilot studies, notably to take into account the inherent differences in viral kinetics among strains/viruses^17,18^. A summary of the two experimental setups performed, for influenza and hRSV/hMPV, respectively, is given in Fig. 1A,B. For the monitoring of viral production and cytokine/chemokine secretion, serial sampling at the apical surface of HAE was performed throughout the time-course of infection. The trans-epithelial electrical resistance (TEER), considered as an indirect marker of the epithelium integrity, was also measured at different time points (gray dots, Fig. 1A,B). Finally, HAE were harvested at 72 hpi (Fig. 1A) or 5 and 6 dpi (Fig. 2A) for RNA extraction and subsequent RTqPCR or high-throughput RNA sequencing.

Influenza viral production was observed at the HAE apical surface as early as 24 hpi, ranging between a minimum of 5.97 log10 TCID50/ml for influenza B and a maximum of 8.8 log10 TCID50/ml for H3N2. Depending on the strain, the viral replication peak was observed between 24 and 72 hpi (Fig. 1C). In the case of hRSV and hMPV infections, significant viral production was measured at the apical surface from 3 dpi, with the viral replication peak being attained between 5 and 6 dpi (Fig. 1D). As a result of these observations of peak viral replication, we selected 72 hpi, 5dpi and 6 dpi as the study end-point for influenza, hMPV and hRSV infections, respectively. Of note, hMPV-B viral titers at the apical surface remained limited even at 5 days post-infection (Fig. 1D). hMPV and hRSV titers at 6 dpi reached 6.42 log10 TCID50/ml and 9.13 log10 TCID50/ml, respectively (Fig. 1D). Trans-epithelial electrical resistance (TEER) values decreased drastically over time in the context of influenza infection (Fig. 1C) but remained stable, similar to that was observed for mock-infected HAE, in the case of hRSV and hMPV (Fig. 1D).

### Differential impact of infection on cytokine/chemokine secretion patterns

To obtain a large overview of the inflammatory and non-inflammatory immune response following HAE infections, we quantified a panel of 27 different cytokines and chemokines using a bead-based multiplex immunoassay (Bio-Plex, Bio-Rad, USA). To facilitate the comparison between experimental conditions, results for each cytokine/chemokine were expressed as the log-transformed ratio compared to mock condition and presented in a radar chart (Fig. 2). All raw data are presented in Supplementary Table 1.

First, the general pattern of cytokine/chemokine secretion compared to mock was relatively similar between the different viruses tested. Indeed, this pattern was notably characterized by the high induction of interleukin 2 (IL-2) and interferon gamma-induced protein 10 (IP-10) in the context of all viral infections studied. These two cytokines, known to be a central part of the pro-inflammatory T helper (Th) 1 response, showed mean relative secretion log-transformed ratios above 2.25 (Fig. 2). This was also the case, though to a slightly lesser extent, for IL-15, with mean secretion log-transformed ratios around 2.25. In addition, the relative level of several cytokines such as IL-5, IL-10, IL-7, IL-13 and Basic-FGF was not as impacted by the infection as the other chemokines/cytokines (Fig. 2). On the other side, we also observed some specificities in the case of hRSV and hMPV. For example, the relative levels of IL-8 and RANTES were increased in all the infections studied, with the notable exception of hMPV. A similar pattern was observed for GM-CSF and hRSV (Fig. 2). In addition, hMPV infection was the only experimental condition tested with a marked increase of VEFG relative level (Fig. 2). Altogether, these results highlight the consistency and reproducibility of the HAE model regarding cytokine secretion upon infection as well as the utility of the generated data for the discrimination between different viral infections.

### Transcriptional profiling of infections in HAE

**Figure 3:**
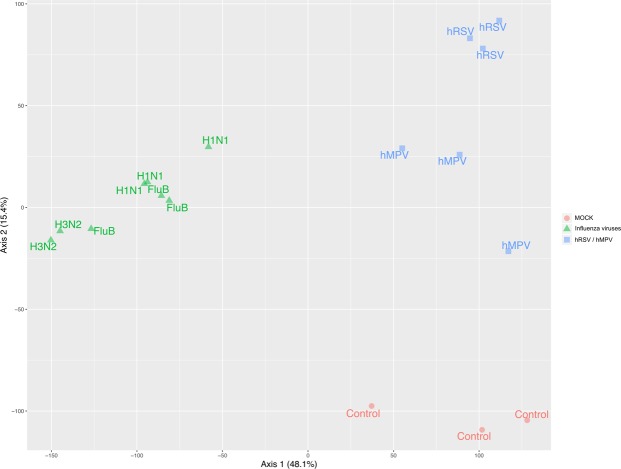
Principal component analysis (PCA) plot depicts significant differences between virus-induced transcriptomic signatures. Abundances in TPM (Transcripts Per Kilobase Million), counts and metadata were combined in a raw expression matrix. HAE infected by viruses belonging to the *Orthomyxoviridae* family (H3N2, H1N1, FluB) are color-coded in green, hMPV and hRSV in blue and mock-infected in red. The component 1 accounts for 48.1% of the total variance of the dataset whereas the second one accounts for 15.4%. The PCA plot showed a distinct progression of differential gene expression mainly between control – influenza and hRSV/hMPV conditions.

For a better understanding of the underlying host response to infection, HAE were lysed at 72 hpi, 5 dpi or 6 dpi and total RNA was extracted. cDNA libraries were then produced, amplified, and subjected to high-throughput sequencing. A Principal Component Analysis (PCA) was performed after data normalization, to assess if the generated data could allow differential clustering between experimental infections and the mock condition. The PCA shows a clear first level of differential clustering between the members of the *Orthomyxoviridae* and *Pneumoviridae* families, but also enables the specific distinction of mock-, Influenza-, hMPV- and hRSV-infected HAE (Fig. 3). Interestingly, differences were tenuous between influenza virus types and subtypes. Based on these PCA clustering results, we postulated that a deeper study of transcriptomic signatures through detailed comparative analysis of differentially modulated genes would provide significant insight on putative inter- and intra-virus family common or distinctive infection features.

### General common and specific features of virogenomic signatures of infection in HAE

**Figure 4:**
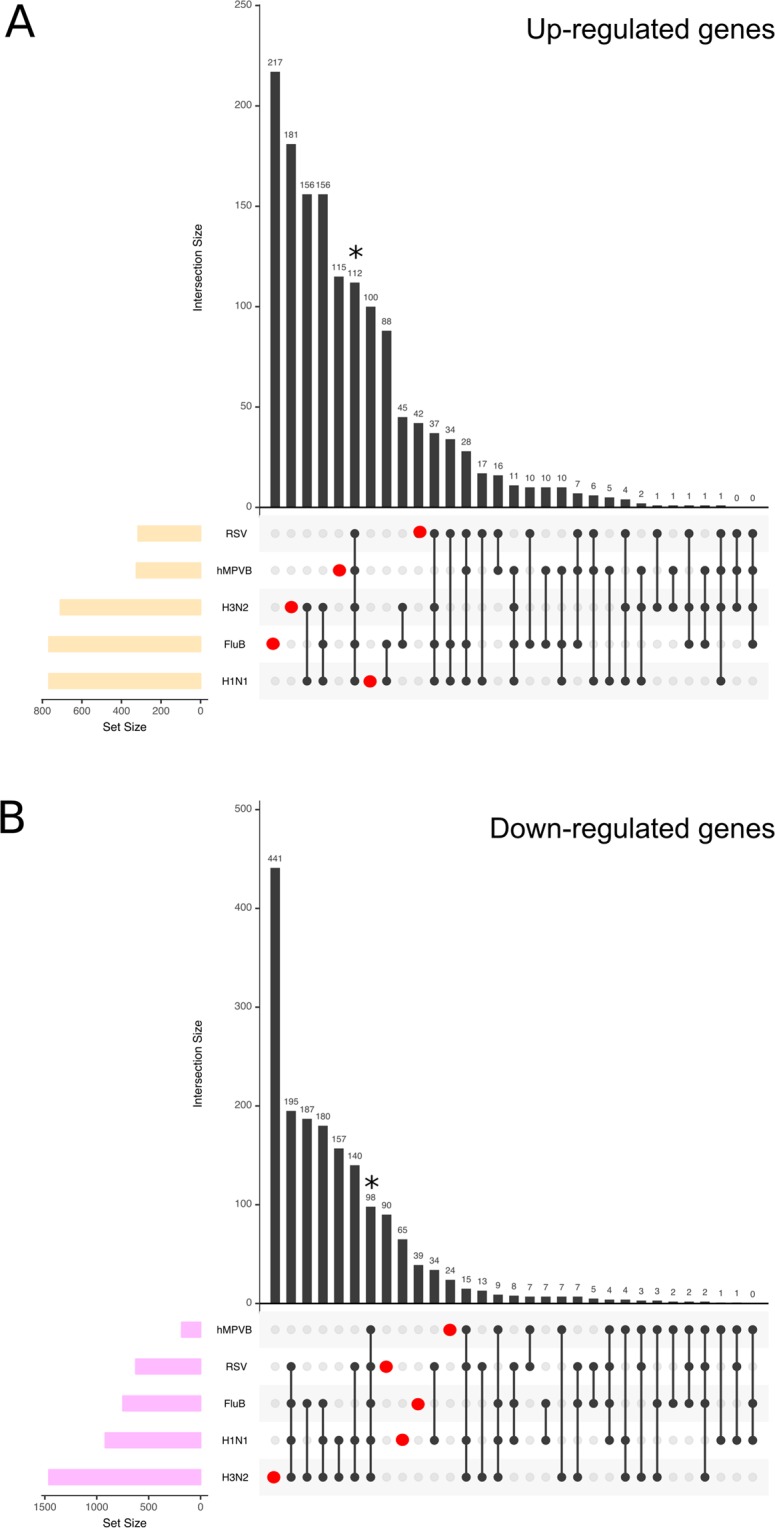
Intersections among host-infected signatures. Differential gene expression was calculated with a p-value threshold <0.05 and an absolute fold-change >2. Intersections among five viral signatures are shown. Panel A accounts for up-regulated genes and panel B for the down-regulated ones. The matrix of solid black or grey circles at the bottom illustrates the “presence” (black circle) or “absence” (grey circle) of the gene sets in each intersection. The numbers to the left of the matrix are set sizes. The colored bars on the top of the matrix represent the intersection sizes. hRSV and hMPV infections account for a lower number of genes differentially expressed and the H3N2 down-regulate the highest number of genes (>1000). 112 and 98 genes (black star) were commonly modulated in all infection studied but each infection also accounted for specific gene modulation (represented in red dots).

Differential expression was calculated by comparing each condition to the mock using a linear model, corrected with the Holm method to control the false discovery rate. Genes with an absolute fold change >2 and a corrected p-value < 0.05 were considered to be differentially expressed. A validation by RT-qPCR was performed on several genes (Supplementary Fig. 1). As shown in Table 1 and Fig. 4 (set size), the number of deregulated genes in the context of hRSV/hMPV infections was lower than that associated with influenza viruses. In addition, whereas the proportion of up-/down-regulated genes was well equilibrated for influenza H1N1 and B viruses, we observed a sharp disequilibrium in favor of up-regulated genes for hMPV, or down-regulated genes for influenza H3N2 and hRSV.

**Table 1:** Number of differentially over- or under-expressed genes compared to mock.

	Genes Up	Genes Down
Influenza A/Lyon/969/2009 (H1N1)	770	918
Influenza A/Texas/50/2012 (H3N2)	709	1461
Influenza B/Massachusetts/2/2012	767	790
hMPV-B strain Can97-82	325	182
hRSV-A strain Long	316	624

Differential expression was calculated by comparing each condition to the mock using a linear model. The Holm procedure was used to control the false discovery rate. Genes with an absolute fold change >2 and a corrected p-value < 0.05 were considered as differentially expressed.

Following this initial observation, we separately analyzed the set of up-regulated (Fig. 4A) and down-regulated genes (Fig. 4B). We first focused our attention on the common set of genes differentially modulated by the 5 viruses studied, which could be considered as the main components of the global virogenomic signature of the infection state (black stars in Fig. 4A,B). This intersection signature consists of a set of 112 up-regulated genes (Fig. 4A) and 98 down-regulated genes (Fig. 4B) that represent around 7.9% and 5.6% of the total number of deregulated genes in the context of infection, respectively. Interestingly, these global signatures represent a large part of the hRSV (more than 37% of up-regulated genes in hRSV-infected HAE) and hMPV (more than 53% of down-regulated genes in hMPV-infected HAE) signatures (Fig. 4 and Table 1). As expected, different influenza viruses share a large set of deregulated genes, notably 156 up-regulated and 180 down-regulated genes. On the other hand, we also investigated the unique sets of genes exclusively modulated by each of the individual viruses tested (red dots in Fig. 4A,B). It is worth noting that these specific sets of genes represented an important proportion of the virogenomic signatures of each virus, particularly in the case of influenza A/Texas/50/2012 (H3N2), in which the number of genes specifically up-regulated (n = 181, Fig. 4A), and down-regulated (n = 441, Fig. 4B) represented almost 29% of the global H3N2 virogenomic signature. Overall, these results suggest that, at least in our experimental conditions, it is possible to identify both common and virus-specific features of how respiratory viral infections impact the cellular transcriptome, even when the compared viruses are very close (*e*.*g*. influenza viruses of the same type).

### Interferon/immune responses and cilium assembly/morphogenesis constitute the hallmarks of viral respiratory infection in HAE

**Figure 5:**
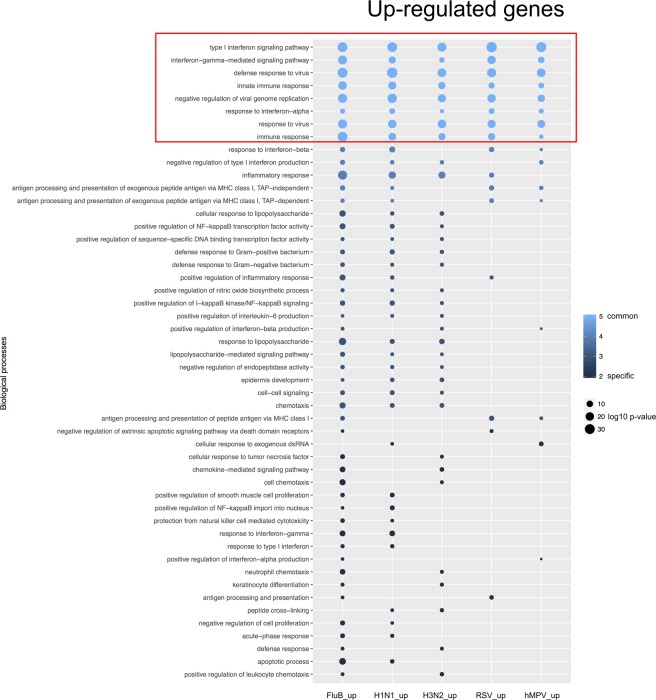
Up regulated genes mainly account for interferon/immune responses in viral-infected HAE. Enrichment was performed on each gene list (up-regulated genes) and biological processes enriched in a least one infection with a Benjamini corrected p-value inferior to 0.01 were plotted. The log10-transformed (Benjamini corrected enrichment p-value) is color-coded in a gradient from dark blue (specific to one infection) to pale blue (common between all five infections). Dot size is proportional to p-value. The absence of dot in a column signifies the absence of enrichment of this specific biological process with the defined thresholds. The only biological processes enriched in every infectious condition immune response related terms (type I interferon signaling pathway, response to virus, (defense) response to interferon-alpha, negative regulation of viral genome replication, interferon-gamma-mediated signaling pathway).

To provide further functional interpretation of the virogenomic signatures in HAE, we then performed a Gene Ontology (GO)-based functional enrichment analysis using the web-based DAVID toolkit. GO terms, and more particularly Biological Processes (BP), were considered enriched when their Benjamini-Hochberg corrected enrichment p-value was <0.01. We first focused on BP enrichment based on the list of up-regulated genes for each virus. Figure 5 shows the enriched BP shared by at least to different viruses. As anticipated, the most enriched BP common to all viruses were associated with Interferon response (IFN alpha - GO:0035455, IFN beta - GO:0035456 and IFN gamma - GO:0060333) and more largely Innate immune response (GO:0045087; GO:0006955) and negative regulation of viral genome (GO:0045071) (Fig. 5). Not surprisingly, a large part of the genes related with these GO terms correlate with the list of 112 up-regulated genes constituting the global virogenomic signatures of infection (black stars in Fig. 4A). Moreover, functional enrichment specific of the group of three influenza viruses studied was observed, notably through BP related to I-kappaB/NF-kappaB signaling (GO:0043123; GO:0042346; GO:0051092) or Apoptotic process (GO:0006915) (Fig. 5).

**Figure 6:**
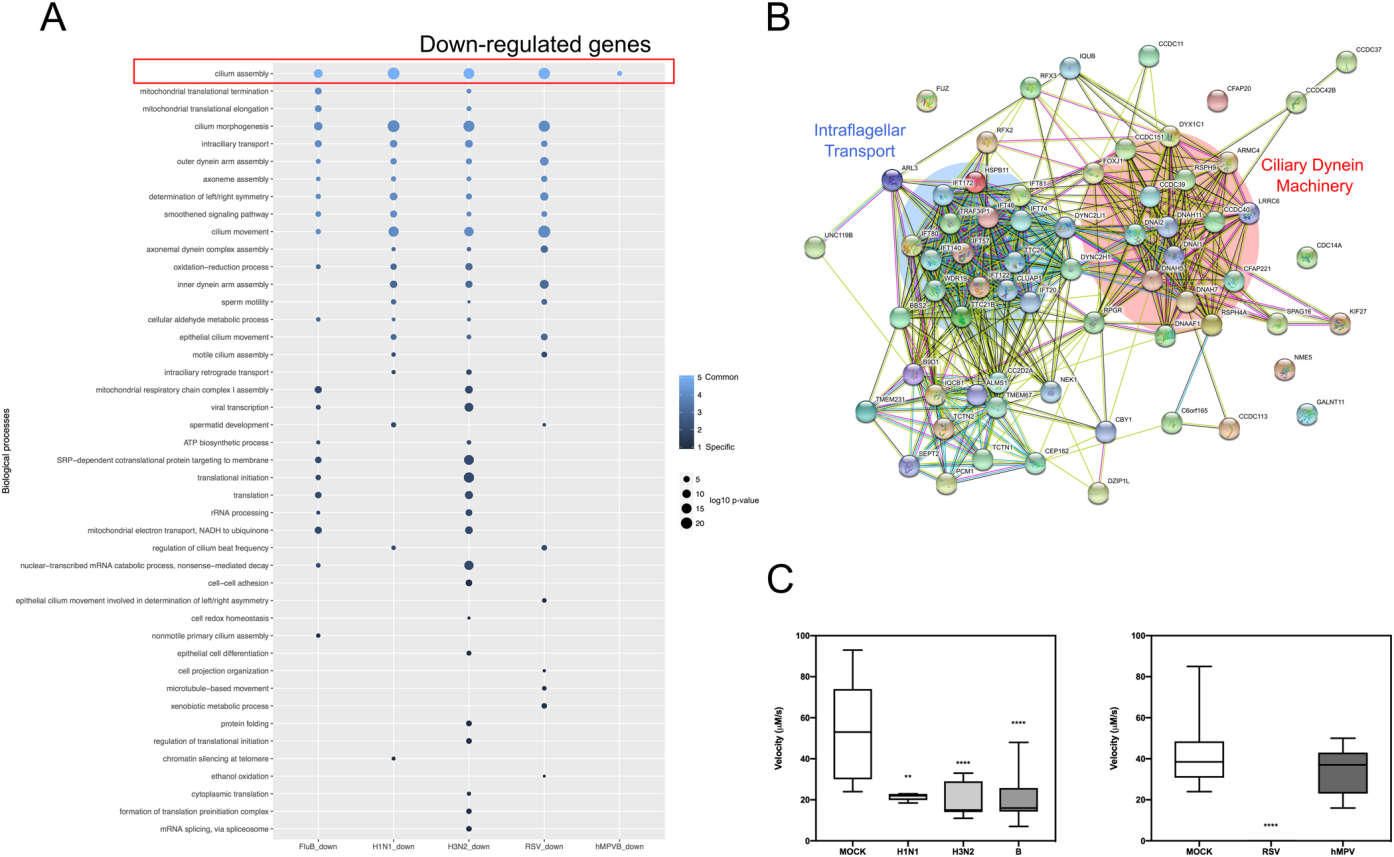
Cilium assembly/morphogenesis is down-regulated in viral-infected HAE. The significant enriched biological processes (down-regulated genes) induced by viral infections and their associated Benjamini-corrected p-values are shown (panel A). The log10-transformed (Benjamini corrected enrichment p-value) is color-coded in a gradient from dark blue (specific to one infection) to pale blue (common between all five infections). Dot size is proportional to p-value. The absence of dot in a column signifies the absence of enrichment of this specific biological process with the defined thresholds. The cilium assembly is common between all infections. A functional network constructed with the STRING database was used to better visualize genes commonly modulated during infections **(**panel B). The two resulting hubs account for proteins related to ciliary dynein machinery and intraflagellar transport. Finally, for an extra-validation of the impact of viral infection on mucociliary clearance, the velocity (panel C) of polystyrene beads was measured at the surface of infected HAE, in the same experimental conditions (MOI, timing) for H1N1, H3N2 and B (left panel), or hRSV hMPV (right panel) and compared to the velocity of identical beads in mock-infected HAE. **p < 0.01 and ***p < 0.0001 compared to MOCK.

In parallel, we also performed an analogous analysis based on the list of down-regulated genes (Fig. 6A). A handful of BPs were identified as being associated with more than one virus, the most enriched BP common to all viruses being Cilium assembly (GO:0042384). Interestingly, other cilium-associated BP such as those related to Cilium morphogenesis or Cilium movement (GO:0060271; GO:0003341) were also enriched in four out of the fifth infections studied (H1N1, H3N2, FluB and hRSV). Most of the genes associated with these GO terms were present either in the list of 98 down-regulated genes constituting the global virogenomic signatures of infection or in the list of 195 down-regulated genes common to influenza viruses and hRSV (Fig. 4B). Interestingly, only the cilium-assembly BP was enriched in the context of hMPV infection, despite a similar number of downregulated genes compared to hRSV, suggesting major differences between these two Pneumoviruses.

To better illustrate the impact of infection on cilium-related BP, we used the list of genes associated to these BP and explored the functional association networks of their protein products using the STRING database^19^ (https://string-db.org). As presented in Fig. 6B, this analysis highlighted a functional network based on 66 distinct proteins (nodes) and 488 protein-protein associations (edges). These associations, which indicate proteins sharing functions (not necessarily physical interaction), are organized in two major hubs concentrating a large number of edges. These two hubs regroup proteins related to ciliary dynein machinery and intraflagellar transport. Altogether, the results of gene enrichment analysis indicate that viral infections strongly impact the cilium-associated cellular machinery, in good agreement with different phenotypic observations. Indeed, beyond the fact that several measures, such as monitoring of TEER (Fig. 1C), suggest that, at least for influenza viruses, infection strongly impacts HAE physiology, we also obtained strong evidence of the negative impact of viral infection on mucociliary clearance. As illustrated in Fig. 6C, using polystyrene microbeads, the mean clearance velocity was reduced by more than 2-fold compared to the Mock condition in the case of influenza H1N1 (p-value < 0.01) and H3N2 and B (p-value < 0.0001) (Fig. 6C, left panel). In the context of hRSV infection, the mucociliary movement was completely abolished (p-value < 0.001), whereas it was only slightly reduced in hMPV-infected HAE (Fig. 6C, right panel). This observation validates a significant reduction of mucociliary clearance consecutive to infection, and is in direct link with the described transcriptional downregulation of the cilium associated cellular machinery.

## Discussion

The main objective of this study was to perform a comparative analysis of transcriptomic signatures of infection among different major respiratory viruses (Influenza, hRSV and hMPV) in HAE. Since these viruses can cause relatively similar respiratory symptoms, it is indeed very interesting to compare their global impact at the level of their primary targets (e.g. respiratory cells) and consecutively better understand similarities and differences between viruses. It is worth noting that only few studies have performed such comparison, most of time limited to two viruses or viruses within the same family (e.g. among IAV subtypes), and with very specific and hence limited readouts (antiviral screening, miRNA profiling, etc)^17,18,20,21^. We advantageously used the HAE Mucilair model, which have been previously used in literature to study viral infections^14–17,22,23^. This experimental model constituted of human primary cells appears as an interesting compromise between the low availability/quantity/quality of clinical samples and the limited biological relevance of immortalized cell lines that are still widely used in fundamental research. A major challenge was to setup experimental conditions allowing a comparison between viruses that present different replicative and infectivity properties. After several setup preliminary experiments, we have defined adapted infection parameters (MOI, timing of infection, sampling time) for each virus in order to study and compare infections at a quite similar stage (during the viral peak). These conditions maximize the extent of HAE cells infected and hence ensure a transcriptional profile that will reflect a global state of infection. Interestingly, despite efficient infection, hRSV and hMPV did not significantly affect TEER values, in contrast with influenza viruses (Fig. 1), suggesting viral-dependent differences of impact on epithelium integrity. In addition we found that hMPV and hRSV viral peaks were reached much later than those of influenza viruses (Fig. 1), which could be also explained by the inherent differences between viruses, but also by the differential mode of viral spreading from one cell to another^24^. Our results suggest that these inherent differences in viral replication kinetics observed in the clinic^25^ are more authentically reproduced in the well-differentiated HAE model when compared to the classic monolayer cell lines widely used in the literature.

Another aim of the study was to couple transcriptomic data with different types of virological, immunological and physiological observations to highlight potential correlations that could help gain better insight from the comparative analysis of the different viruses studied. The use of the HAE model allowed us to monitor several parameters at comparable time points and to investigate possible correlations between phenotypic observations/physiological measures and virogenomic signatures. For example, we demonstrated that the viral peak was correlated with a marked decrease of TEER values and changes on the epithelial surface visible through optical microscopy in the case of influenza viruses (Fig. 1C), in good agreement with previously published observations by our group^26^. This inverse correlation could be linked with the strong negative impact of influenza viruses on the regulation of cilium-associated genes (Fig. 6), with a consecutive impact on epithelium integrity. The major enrichment of down-regulated cilium-associated genes observed was also clearly associated to a strong decrease of mucociliary clearance in the context of infection (Fig. 6C). Another interesting transversal observation was made at the cytokine level. Indeed, the retrospective analysis of our virogenomic profiles indicate a relatively good level of concordance between the regulation of genes coding cytokines/chemokines and the measure of cytokines by a multiplex immunoassay, as exemplified by IP-10 (and to a lesser extent RANTES, IL-6, MIP-1B, IL-1B or IL1-RA), in line with previous observations^27^ (Fig. 2, Supplementary Fig. 1 and Table 2).

The comparative analysis of transcriptional profiles generated in this study allowed us to establish a common global virogenomic signature of infection, constituted by a subset of 112 up-regulated and 98 down-regulated genes (Fig. 4A,B) that reflect the host response to the different respiratory viruses. Interestingly, we found that, despite being relatively distinct in many aspects of their infection cycle and pathogenesis, viruses such as hMPV and influenza viruses share a large common signature, mostly through the upregulation of genes directly or indirectly involved in the IFN response (Fig. 5), and notably the type III (lambda) IFN response (Supplementary Fig. 1). Type III is major IFN known to be secreted by the human respiratory epithelium following influenza and hRSV infections *in vivo*^28,29^, which also illustrates the biological relevance of the HAE experimental model used in this study. In contrast, less information is available regarding the IFN response in hMPV-infected individuals, but some reports suggest that hMPV infection also induces type III IFN *in vivo*, yet at lower levels in the case of premature infants^30,31^. Whereas the nature of the viral inducers of this type III IFN response in HAE could (or not) be different between influenza, hRSV and hMPV, this response most certainly occurs *via* the same ligands and signaling components, a phenomenon that could be considered of great interest especially in the context of viral co-infections and also for the design of broad-spectrum antiviral therapeutic strategies. Another interesting common feature revealed by the global virogenomic signature shared between hRSV, hMPV and influenza viruses is the global downregulation of cilium-related genes, mostly related to intraflagellar transport and ciliary dynein machinery (Fig. 6B), whose protein products are critical for the assembly, maintenance and function of motile and sensory cilia^32^. These transcriptomic data were in good agreement with the indirect evaluation of mucociliary clearance, using polystyrene microbeads (Fig. 6C). The mucociliary clearance is one of the first and foremost, though often overlooked, lines of defense of the epithelium against infection^33^. Indeed, a relatively limited amount of data, mostly obtained in studies dedicated to influenza viruses and hRSV, is available regarding the impact of respiratory viruses on the epithelium cilia. More particularly, the impact of respiratory viral infections on cilium-related gene expression was not specifically investigated. Several reports indicate that influenza infection in airway epithelial experimental models results in a loss of ciliated cells and the associated impairment of mucociliary clearance, which could explain why a primary viral infection can “pave the way” for a secondary infection by other pathogens such as bacteria^34,35^. In addition, hRSV has also been reported to induce an increase on ciliary dyskinesis combined with ciliary loss and epithelial damage, hence resulting in a strong decrease of mucociliary clearance^36^. This interplay between respiratory viruses and the cilium-related machinery is of great interest, even more in the context of pathological defective mucociliary clearance such as cystic fibrosis, where co-infections are known to be associated with more severe respiratory pathologies^37,38^.

In conclusion, our descriptive study using a comparative transversal approach in a HAE model highlights several facets of the host responses that are common/specific to the infection process by different major respiratory viruses. Future investigations will focus more precisely on the specific virogenomic profile of hMPV in HAE, which appears to be relatively distinct to the other viruses studied. In addition, these collected data constitute a very interesting starting point for future studies focused on specific cellular factors, sometimes overlooked in previous studies performed in less relevant cellular models. Our work also indicates that it is possible to combine different types of experimental approaches and parameters in order to discriminate the etiology of respiratory viral infections with very similar clinical manifestations. In that regard, further development in this direction would be promising for the future characterization of new biomarkers or groups of biomarkers of diagnostic interest, or as surrogates for the efficacy evaluation of future innovative treatments. Finally, the virogenomic signatures of infection determined in this study could also be used for the future identification and repurposing of molecules for their broad-spectrum antiviral activities^26^ and/or alternatively, the identification of new cellular targets of choice for the design of new host-targeted therapeutic approaches.

## Materials and Methods

### Cells and viruses

Influenza A/Lyon/969/2009(H1N1) (2009 pandemic strain), B/Massachusetts/2/2012 and A/Texas/50/2012(H3N2) viral strains were produced in MDCK cells (ATCC CCL-34), using EMEM supplemented with 2 mM L-glutamine (Sigma Aldrich), penicillin (100 U/mL), streptomycin (100 μg/mL) (Lonza) and 1 µg/mL Lys-acetylated bovine pancreas trypsin at 37 °C and 5% CO2. Viral titers in plaque forming units (PFU/ml) and tissue culture infectious dose 50% (TCID50/mL) were determined in MDCK cells as previously described^39,40^. Respiratory syncytial virus (hRSV-A Long strain ATCC-VR26) and human metapneumovirus (hMPV-B strain CAN97-82 (B1) and CAN98-75 (B2)) were produced in Hep-2 cells (ATCC CCL-23) and in LLCMK2 cells (ATCC CCL7), respectively, using EMEM supplemented with 2 mM L-glutamine (Sigma Aldrich), penicillin (100 U/mL), streptomycin (100 μg/mL) (Lonza), at 37 °C and 5% CO2. Viral stocks were titrated in tissue culture infectious dose 50% (TCID50/mL) in the case of hRSV and in fluorescent focus units (ffu/mL) in the case of hMPV, as previously described^41^.

### Viral infection in reconstituted human airway epithelium (HAE)

MucilAir HAE, reconstituted using human primary cells obtained from nasal biopsies, were purchased from Epithelix SARL (Geneva, Switzerland) and maintained in air-liquid interphase with specific culture medium in Costar Transwell inserts (Corning, NY, USA) according to the manufacturer’s instructions. For infection experiments, apical poles were gently washed twice with warm OptiMEM medium (Gibco, ThermoFisher Scientific) and then infected with a 150 µL dilution of the different viruses in OptiMEM medium, at a multiplicity of infection (MOI) of 0.1 (hMPV-B CAN97-82 and influenza B/Massachusetts/2/2012), 1 (RSV-A Long) or 10 (influenza A/Lyon/969/2009(H1N1) and influenza A/Texas/50/2012(H3N2)). After 1 h incubation at 37 °C and 5% CO2, the viral suspension was removed and HAE were again gently washed with 300 µL of OptiMEM. For mock infection, the same procedure was performed using OptiMEM as the inoculum. For viral kinetics studies, apical sampling was performed between 24- and 72-hours post-infection (hpi) for influenza viruses, or at 3 and 5- or 6-days post-infection (dpi) respectively for hMPV and RSV. Collected samples were separated into 2 tubes: one for viral titration and one for cytokine assay. Variations in trans-epithelial electrical resistance (Δ TEER) were measured using a dedicated volt-ohm meter (EVOM2, Epithelial Volt/Ohm Meter for TEER) and expressed as Ohm/cm^2^. At the end of viral kinetics (72 hpi, 5 dpi or 6 dpi), cells were harvested in RLT buffer (Qiagen) and total ARN was extracted using the RNeasy Mini Kit (Qiagen). For indirect evaluation of mucociliary clearance, 10μm-diameter polystyrene microbeads were seeded onto the apical surface of HAE. The particle movement was tracked using videomicroscopy and ImageJ software and was expressed as a velocity (μm/s).

### Multiplex immunoassay of cytokines/chemokines

Samples collected at apical poles of HAE were screened for the presence of 27 human cytokines and chemokines, as previously described^27^, using the Bio-Plex Pro Human Cytokine Standard 27-Plex kit (Bio-Rad, Hercules, California, USA) on a FLEXMAP 3D analyzer (Luminex, Austin, Texas, USA). Concentrations outside the observation range were arbitrary replaced by zero (if the observation is reported below the detection threshold) or 800000 (if the observation was above the detection threshold). Mean signal was calculated for each condition. For comparison purpose, for each cytokine/chemokine, results were expressed as log-transformed ratio compared to Mock except for the RSV GM-CSF condition where no signal could be measured for which it was set to zero. Visualization of results was achieved using the fmsb R package.

### Real-time quantitative PCR

Each HAE was lysed with 150 µl of RLT buffer (Qiagen) 72 h p.i, 5dpi or 6dpi for influenza viruses, hMPV and hRSV, respectively. Total RNA was extracted using the RNeasy Mini Kit (Qiagen) according to the manufacturer’s instructions. After reverse transcription, real-time qPCR was performed using the StepOnePlus™ Real-Time PCR System (Applied Biosystems) in 96-well plates. qPCR primers (GAPDH: Hs02758991_g1, IFNL1: Hs00601677_g1, IFNL2: Hs00820125_g1, IFIT2: Hs00533665_m1,, IFI44L: Hs00915292_m1) and probe (TaqMan gene expression assays) were provided by Thermo Fisher Scientific. GAPDH was included in each well, in duplex, as the endogenous standard reference. Each sample was analyzed in duplicate, and the cycle threshold (Ct) values were normalized against the endogenous GAPDH reference. Relative changes in gene expression were determined using the ΔΔCt method and reported as the fold change relative to the uninfected mock control.

### Sample processing, RNA preparation and sequencing

The Scriptseq complete Gold kit-Low Input (SCL6EP, Epicentre) was used according to manufacturer’s instructions to prepare cDNA libraries from 200 ng of total RNA. Amplified and indexed libraries with primers provided in the ScriptSeq Index PCR Primers kit (RSBC10948, Epicentre) were quantified with QuBit and Bioanalyzer2100, pooled in equimolar concentrations and sequenced as 100 bp paired-end reads on an Illumina HiSeq. 2500 system (Illumina, Carlsbad, CA), with a required minimum of 40 million reads sequenced per sample. Conversion and demultiplexing of reads were performed using bcl2fastq 1.8.4 (Illumina). The FastQC software (http://www.bioinformatics.babraham.ac.uk/projects/fastqc) was used for quality controls of the raw data. Reads were trimmed using the Trimmomatic software^42^, with a minimum quality threshold of Q30. Reads were pseudo-aligned to the Homo sapiens genome (GRCh38.p11) using the Kallisto software^43^. The results of the main quantification (abundance estimate in Transcripts Per Million) were used for principal component analysis (PCA) with the R packages factoextra and FactoMineR^44^. Statistical analysis was performed in R3.3.1 with the package EdgeR 3.14.0^45^. Differential expression was calculated by comparing each condition to the mock using a linear model. The Holm procedure was used to control the false discovery rate. Genes with an absolute fold change >2 and a corrected p-value < 0.05 were considered to be differentially expressed. Visualization of subsequent analysis results was achieved using the UpsetR and ggplot2 R packages.

### Functional analysis

Pathway and functional enrichment analysis were carried out using the web-based tool DAVID v6.8 (Database for Annotation, Visualization, and Integrated Discovery)^46^. GO terms and pathways were considered enriched when their Benjamini-Hochberg corrected enrichment p-value was below 0.01.

## Supplementary information

Supplementary Figure 1

Supplementary Table 1

Supplementary Table 2
