# Supplementary material for: Characterization of cellular transcriptomic signatures induced by different respiratory viruses in human reconstituted airway epithelia
Source: Sci Rep. 2019 Aug 7;9:11493. doi: 10.1038/s41598-019-48013-7 (PMC6685967; doi:10.1038/s41598-019-48013-7)
Supplement: Supplementary file 1 — Supplementary Figure 1 [file 41598_2019_48013_MOESM1_ESM.docx]

**Title**

Characterization of cellular transcriptomic signatures induced by different respiratory viruses in human reconstituted airway epithelia

**Authors**

Claire Nicolas de Lamballerie, Andrés Pizzorno, Julia Dubois, Thomas Julien, Blandine Padey, Mendy Bouveret, Aurélien Traversier, Catherine Legras-Lachuer, Bruno Lina, Guy Boivin, Olivier Terrier and Manuel Rosa-Calatrava


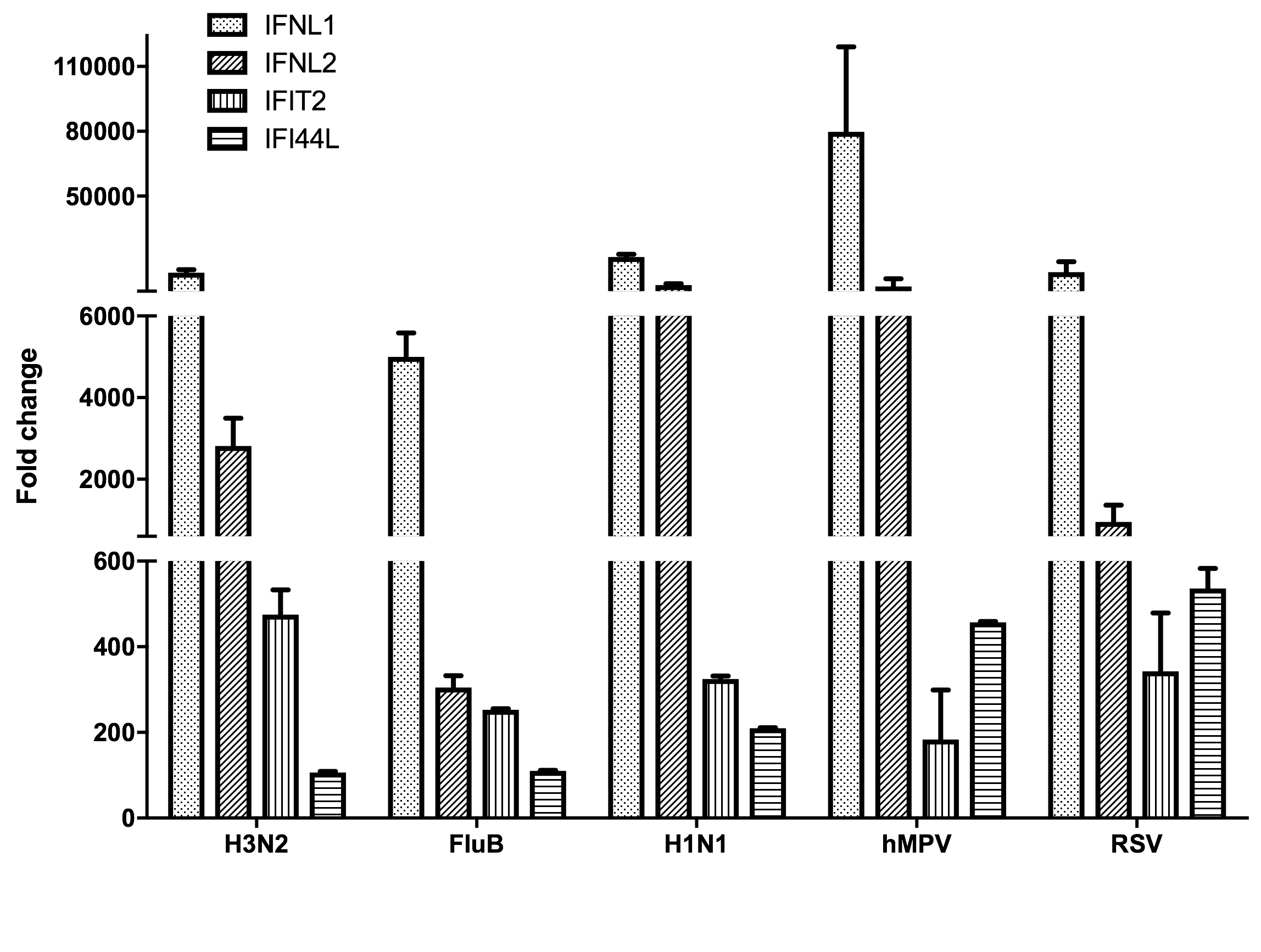


**Supplementary Figure 1. RT-QPCR validation**

Each HAE was lysed with 150 µl of RLT buffer (Qiagen) 72 h p.i, 5dpi or 6dpi for influenza viruses, hMPV and hRSV, respectively. Total RNA was extracted using the RNeasy Mini Kit (Qiagen) according to the manufacturer’s instructions. After reverse transcription, real-time qPCR was performed using the StepOnePlus™ Real-Time PCR System (Applied Biosystems) in 96-well plates. qPCR primers (GAPDH: Hs02758991_g1, IFNL1: Hs00601677_g1, IFNL2: Hs00820125_g1, IFIT2: Hs00533665_m1, IFI44L: Hs00915292_m1) and probe (TaqMan gene expression assays) were provided by Thermo Fisher Scientific. GAPDH was included in each well, in duplex, as the endogenous standard reference. Each sample was analyzed in duplicate, and the cycle threshold (Ct) values were normalized against the endogenous GAPDH reference. Relative changes in gene expression were determined using the ΔΔCt method and reported as the fold change relative to the uninfected mock control.
