# Supplementary material for: Characterization of cellular transcriptomic signatures induced by different respiratory viruses in human reconstituted airway epithelia
Source: Sci Rep. 2019 Aug 7;9:11493. doi: 10.1038/s41598-019-48013-7 (PMC6685967; doi:10.1038/s41598-019-48013-7)
Supplement: Supplementary file 2 — Supplementary Table 1 [file 41598_2019_48013_MOESM2_ESM.pdf]

Title

Characterization of cellular transcriptomic signatures induced by different respiratory viruses in human reconstituted airway epithelia

Authors

Claire Nicolas de Lamballerie, Andrés Pizzorno, Julia Dubois, Thomas Julien, Blandine Padey, Mendy Bouveret, Aurélien Traversier, Catherine Legras-Lachuer, Bruno Lina, Guy Boivin, Olivier Terrier and Manuel Rosa-Calatrava

Supp Table 1.

	Basic FGF (44)	Eotaxin (43)	G-CSF (57)	GM-CSF (34)	IFN-G (21)	IL-10 (56)	IL-12P70 (75)	IL-13 (51)	IL-15 (73)	IL-17 (76)
Description	Obs Conc	Obs Conc	Obs Conc	Obs Conc	Obs Conc	Obs Conc	Obs Conc	Obs Conc	Obs Conc	Obs Conc
MOCK	669,77	920,2	5307,26	0	619,47	419,15	852,79	129,1	0	976,82
MOCK	704,03	778,15	2586,7	0	774,11	565,96	834,98	95,13	0	684,68
MOCK	520,66	1244,1	6327,25	0	1175,18	433,82	687,53	156,43	6,33	684,68
H1N1	910,63	4004,15	40928,85	0	6898,13	737,56	1308,24	246,68	488,86	3267,99
H1N1	794,67	3748,68	41287,12	26,52	6554,2	624,91	1379,67	254,91	380,89	3232,86
H1N1	827,87	3840,28	39817,77	152,34	6496,12	462,67	1134,51	238,19	311,66	3302,83
H3N2	879,95	4466,42	46805,55	860,06	8673,34	663,17	1711,29	201,09	594,56	4919,53
H3N2	980,8	4573,8	66596,77	777,43	8877,44	924,8	1795,5	238,19	587,05	4848,17
H3N2	934,84	4509,72	54571,98	672,49	8801,14	1067,82	2311,23	238,19	529,87	4296,27
B	849,63	3722,04	42915,05	377,82	6612,07	761,79	1596,86	129,1	496,74	3827,56
B	801,04	3338,75	29578,58	313,63	5280,5	700,7	1730,12	180,01	331,51	3152,76
B	889,13	3529,17	38120,86	226,92	6291	809,48	2031,96	201,09	420,43	3439,57
hRSV	617,65	3086,03	8860,92	0	4625,06	1385,74	3394,44	278,29	397,66	2390,85
hRSV	688,53	3035,99	12179,77	0	4420,49	1375,9	2807,65	201,09	408,05	2562,93
hRSV	680,58	3052,77	12994,85	0	5055,36	1385,74	3076,4	313,75	516,3	2766,62
hMPVB	851,57	4488,12	10665,03	1581,9	8100,83	3280,77	6700,82	477,73	1368,47	3995,87
hMPVB	775,09	4455,52	8755,76	1710,58	8258,68	2898,67	6201,46	397,91	1136,01	4070,65

	IL-1B (39)	IL-1RA (25)	IL-2 (38)	IL-4 (52)	IL-5 (33)	IL-6 (19)	IL-7 (74)	IL-7 (74)	IL-8 (54)	IL-9 (77)
Description	Obs Conc	Obs Conc	Obs Conc	Obs Conc	Obs Conc	Obs Conc	Conc in Rang	Obs Conc	Obs Conc	Obs Conc
MOCK	8,3	821,72	0	54,74	0	4042,23	1205,78	1205,78	24582,38	979,04
MOCK	6,9	665,84	0	79,3	0	2142,26	2036,63	2036,63	19239,82	980,92
MOCK	8,3	1090,83	0	84,46	122,64	4722,26	1419,78	1419,78	24598,63	1048,8
H1N1	131,05	10382,93	634,18	329,24	0	32102,79	1703,54	1703,54	OOR >	2723,58
H1N1	128,38	12012,88	649,27	335,39	0	31812,1	2802,16	2802,16	OOR >	2731,23
H1N1	132,5	10132,54	560,1	319,83	160,71	29413,88	459,71	459,71	OOR >	2594,62
H3N2	251,95	22354,08	872,72	401,54	64,63	OOR >	2609,12	2609,12	OOR >	3672,07
H3N2	386,18	18969,64	852,27	404,15	160,71	42980,38	2114,01	2114,01	OOR >	3472,61
H3N2	331,45	16839,42	852,27	419,5	160,71	43542,53	2609,12	2609,12	OOR >	3737,06
B	176,69	14417,54	753,31	359,08	64,63	33003,77	2864,5	2864,5	OOR >	3118,48
B	97,98	6489,94	484,56	288,29	64,63	23863,91	1790,88	1790,88	OOR >	2596,59
B	134,42	8089,96	603,21	311,79	122,64	27745,2	2189,45	2189,45	OOR >	2843,83
hRSV	54,02	2828,04	378,25	262,06	122,64	16103,73	3552,1	3552,1	OOR >	1739,99
hRSV	46,41	2060,49	453,23	255,39	191,24	16013,3	3105,12	3105,12	OOR >	1830,83
hRSV	52,67	2960	370,92	268,6	217,47	21004,99	3863,9	3863,9	OOR >	1767,32
hMPVB	72,42	5968,48	892,85	420,13	325,5	29832,85	9605,29	9605,29	OOR >	2431,72
hMPVB	88,37	6600,19	827,26	430,09	378,31	34799,67	8969,54	8969,54	41716,9	2351,6

	IP-10 (48)	MCP-1 (53)	MIP-1A (55)	MIP-1B (18)	PDGF-BB (47)	RANTES (37)	TNF-A (36)	VEGF (45)
Description	Obs Conc	Obs Conc	Obs Conc	Obs Conc	Obs Conc	Obs Conc	Obs Conc	Obs Conc
MOCK	18877,15	2969,56	0	161,46	OOR <	216,04	111,53	8005,55
MOCK	11338,66	2160,91	16,2	93,05	315,78	113,93	87,27	6972,95
MOCK	25802,06	1906,37	46,8	144,03	0	295,77	60,38	7136,37
H1N1	OOR >	7909,3	344,05	2957,64	2123,71	21316,33	1499,66	12222,17
H1N1	OOR >	6056,74	334,9	3045,04	2326,86	20874,2	1304,15	11011,2
H1N1	OOR >	5506,15	337,7	3021,66	2048,1	18298,96	1386,76	8196,39
H3N2	OOR >	6465,1	878,01	7778,95	3398,22	32600,58	2370,5	12543,8
H3N2	OOR >	6407,3	961,19	6704,17	2797,63	18915,51	3091,39	15916,87
H3N2	OOR >	6702,16	896,11	6276,97	2901,98	18555,07	2213,63	18109,79
B	OOR >	7277,78	664,75	4622,73	2541,36	12970,55	4262,57	15190
B	OOR >	5532,71	347,16	2824,16	1992,42	8086,5	2839,45	13976,84
B	OOR >	7532,42	492,29	3301,94	2037,08	8410,75	3241,65	14352,69
hRSV	OOR >	5008,13	87,72	919,83	3735,38	8197,39	711,03	36131,11
hRSV	OOR >	6863,47	89,02	883,55	3266,8	7154,76	652,61	31771,88
hRSV	OOR >	5225,08	86,4	814,56	4214,96	7056,93	702,79	31346,98
hMPVB	OOR >	14630,28	111,94	495,54	19192,38	3707,39	1105,04	OOR >
hMPVB	OOR >	12368,78	118,31	478,63	19142,44	2839,5	1105,04	OOR >
