# Supplementary material for: Characterization of cellular transcriptomic signatures induced by different respiratory viruses in human reconstituted airway epithelia
Source: Sci Rep. 2019 Aug 7;9:11493. doi: 10.1038/s41598-019-48013-7 (PMC6685967; doi:10.1038/s41598-019-48013-7)
Supplement: Supplementary file 3 — Supplementary Table 2 [file 41598_2019_48013_MOESM3_ESM.pdf]

Title

Characterization of cellular transcriptomic signatures induced by different respiratory viruses in human reconstituted airway epithelia

Authors

Claire Nicolas de Lamballerie, Andrés Pizzorno, Julia Dubois, Thomas Julien, Blandine Padey, Mendy Bouveret, Aurélien Traversier, Catherine Legras-Lachuer, Bruno Lina, Guy Boivin, Olivier Terrier and Manuel Rosa-Calatrava

Supp. Table 2

Entrez		Cytokine	Genes	H1N1	FluB	H3N2	hMPVB	RSV	alias
2247	2247	**Basic FGF**	*FGF2*						BFGF, FGF-2, FGFB, HBGF-2, fibroblast growth factor 2
		**Eotaxin**							
6356	6356		CCL11						SCYA11, C-C motif chemokine ligand 11
6369	6369		CCL24						SCYA24, Ckb-6, MPIF-2, eotaxin-2
10344	10344		CCL26						SCYA26, MIP-4alpha, eotaxin-3, IMAC, MIP-4a, TSC-1
1440	1440	**G-CSF**	CSF3		249,44	62,93			C17orf33, CSF3OS, GCSF, colony stimulating factor 3
1437	1437	**GM-CSF**	CSF2		43,36	24,47			GMCSF, colony stimulating factor 2, CSF
3458	3458	**IFN-G**	IFNG						IFG, IFI, interferon, gamma, interferon gamma
3586	3586	**IL-10**	IL10						CSIF, GVHDS, IL-10, IL10A, TGIF, interleukin 10
3592	3592	**IL-12P70**	IL12A						CLMF1, NKSF1, p35
3593	3593		IL12B						CLMF2, NKSF2, p40
3596	3596	**IL-13**	IL13						IL-13, P600, interleukin 13
3600	3600	**IL-15**	IL15						IL-15, interleukin 15
		**IL-17**							
3605	3605		IL17A						IL17, CTLA8
27190	27190		IL17B						ZCOTO7
271989	271989		IL17C						CX2
53342	53342		IL17D						
3553	3553	**IL-1B**	IL1B	12,01	78,19	18,95			IL-1, IL1-BETA, IL1F2, interleukin 1 beta
3557	3557	**IL-1RA**	IL1RN	9,01	75,21	15,25		3,74	DIRA, ICIL-1RA, IL-1RN, IL-1ra, IL-1ra3, IL1F3, IL1RA, IRAP, MVCD4, interleukin 1 receptor antagonist
3558	3558	**IL-2**	IL2						IL-2, TCGF, lymphokine, interleukin 2
3565	3565	**IL-4**	IL4						BCGF-1, BCGF1, BSF-1, BSF1, IL-4, interleukin 4
3567	3567	**IL-5**	IL5						EDF, IL-5, TRF, interleukin 5
3569	3569	**IL-6**	IL6	28,18	165,44	41,66			BSF2, HGF, HSF, IFNB2, IL-6, BSF-2, CDF, IFN-beta-2, interleukin 6
3574	3574	**IL-7**	IL7						IL-7, interleukin 7
3576	3576	**IL-8**	IL8		21,95				chemokine (C-X-C motif) ligand 8, GCP-1, GCP1, LECT, LUCT, LYNAP, MDNCF, MONAP, NAF, NAP-1, NAP1, IL8, C-X-C motif chemokine ligand 8, Interleukin-8
3578	3578	**IL-9**	IL9						HP40, IL-9, P40, interleukin 9
3627	3627	**IP-10**	CXCL10	742,94	1363,64	310,17	103,18	211,55	C7, IFI10, INP10, IP-10, SCYB10, crg-2, gIP-10, mob-1, C-X-C motif chemokine ligand 10, C-X-C motif chemokine 10
6347	6347	**MCP-1**	CCL2		7,40				GDCF-2, HC11, HSMCR30, MCAF, MCP-1, MCP1, SCYA2, SMC-CF, C-C motif chemokine ligand 2
6348	6348	**MIP-1A**	CCL3		136,95				G0S19-1, LD78ALPHA, MIP-1-alpha, MIP1A, SCYA3, C-C motif chemokine ligand 3
6351	6351	**MIP-1B**	CCL4	55,55	700,60	131,65			ACT2, AT744.1, G-26, HC21, LAG-1, LAG1, MIP-1-beta, MIP1B, MIP1B1, SCYA2, SCYA4, C-C motif chemokine ligand 4
5155	5155	**PDGF-BB**	PDGFB						IBGC5, PDGF-2, PDGF2, SIS, SSV, c-sis, platelet derived growth factor subunit B
6352	6352	**RANTES**	CCL5	304,41	397,57	367,83		48,26	D17S136E, RANTES, SCYA5, SIS-delta, SISd, TCP228, eoCP, C-C motif chemokine ligand 5
7124	7124	**TNF-A**	TNF	21,51	104,13	43,77			DIF, TNF-alpha, TNFA, TNFSF2, Tumour necrosis factor, TNF-α, tumor necrosis factor, TNLG1F, Tumor necrosis factor alpha
		**VEGF**							
7422	7422		VEGFA		5,13				Vascular Endothelial Growth Factor A
7423	7423		VEGFB						Vascular Endothelial Growth Factor B
7424	7424		VEGFC						Vascular Endothelial Growth Factor C
